# Insights into miRNAs of the Stingless Bee *Melipona quadrifasciata*

**DOI:** 10.3390/ncrna11030048

**Published:** 2025-06-19

**Authors:** Dalliane Oliveira Soares, Lucas Yago Melo Ferreira, Gabriel Victor Pina Rodrigues, João Pedro Nunes Santos, Ícaro Santos Lopes, Lucas Barbosa de Amorim Conceição, Tatyana Chagas Moura, Isaque João da Silva de Faria, Roenick Proveti Olmo, Weyder Cristiano Santana, Marco Antônio Costa, Eric Roberto Guimarães Rocha Aguiar

**Affiliations:** 1 Department of Biological Sciences, Center of Biotechnology and Genetics, Universidade Estadual de Santa Cruz (UESC), Ilhéus 45662-900, Brazil; dalli.biotec@gmail.com (D.O.S.); lucasmelobiomed@gmail.com (L.Y.M.F.); gvprodrigues.ppggbm@uesc.br (G.V.P.R.); jpnsantos.bio@uesc.br (J.P.N.S.); lbaconceicao.ppggbm@uesc.br (L.B.d.A.C.); 2 Department of Biochemistry and Immunology, Universidade Federal de Minas Gerais, Belo Horizonte 31270-901, Brazil; icarolopes.bio@gmail.com (Í.S.L.); tatychagas1@gmail.com (T.C.M.); isaquejsf@gmail.com (I.J.d.S.d.F.); costama@uesc.br (M.A.C.); 3 Centre National de la Recherche Scientifique (CNRS) UPR9022, Inserm U1257, Institut de Biologie Moléculaire et Cellulaire, 16 CEDEX, 67084 Strasbourg, France; roenick@gmail.com; 4 Department of Entomology, Universidade Federal de Viçosa, Viçosa 36570-900, Brazil; weyder.santana@ufv.br; 5 Postgraduate Program in Computational Modeling in Science and Technology, Department of Engineering and Computing, State University of Santa Cruz (UESC), Ilhéus 45662-900, Brazil

**Keywords:** gene regulation, Hymenoptera, Meliponini miRNA, non-coding RNAs, pollinator genomics

## Abstract

MicroRNAs (miRNAs) are key post-transcriptional regulators involved in a wide range of biological processes in insects, yet little is known about their roles in stingless bees. Here, we present the first characterization of miRNAs in *Melipona quadrifasciata* using small RNAs (sRNAs) deep sequencing. A total of 193 high-confidence mature miRNAs were identified, including 106 *M. quadrifasciata*-exclusive sequences. Expression profiling revealed that mqu-miR-1 and mqu-miR-276 together accounted for over 70% of all miRNA reads, suggesting their central roles in development and reproduction. Comparative analyses showed a higher conservation of *M. quadrifasciata* miRNAs with other Hymenopterans, especially *Apis mellifera* and *Bombus* spp. Putative target genes were predicted using a consensus approach, and functional annotation indicated their involvement in diverse biological regulatory pathways. This work represents the first comprehensive identification of the miRNA repertoire in stingless bees using sRNAs and provides a valuable foundation for understanding miRNA-mediated gene regulation in this ecologically and economically important pollinator.

## 1. Introduction

*Melipona quadrifasciata* Lepeletier (Hymenoptera: Apidae), commonly known as mandaçaia, is a Brazilian stingless bee of great ecological and economic relevance that stands out for its ability to adapt to urban and agricultural environments [1]. This species plays a crucial role in pollination, which is essential for about one-third of global food production, and contributes hundreds of billions of dollars annually to agriculture [2,3]. Behaviors such as buzz pollination and its ability to forage on a wide variety of plants make mandaçaia an efficient pollinator of crops like tomato, coffee, apple, and guava [4,5,6]. However, its survival is threatened by factors such as pathogens, especially viruses, climate change, habitat loss, and excessive pesticide use, all of which impact its health, behavior, and distribution [7,8,9,10], highlighting the urgent need for conservation strategies.

RNA interference (RNAi) stands out as a key sequence-specific, post-transcriptional gene silencing mechanism [11,12]. This defense is mediated by small RNAs, including miRNAs and small interfering RNAs (siRNAs), which play fundamental roles in gene regulation. In addition to their involvement in antiviral defense, miRNAs have been associated with key processes in bees such as caste determination, responses to environmental stressors [13], the regulation of complex social behaviors [14], and defense against parasites like *Nosema ceranae* Fries (Microsporidia: Nosematidae) [15].

Although miRNAs have been widely studied in model organisms like *Drosophila melanogaster* Meigen (Diptera: Drosophilidae) and *Apis mellifera* Linnaeus (Hymenoptera: Apidae), we still know little about these important regulators in stingless bees (tribe Meliponini). We believe that miRNAs in stingless bees have unique expression patterns and regulatory roles that help explain their evolutionary success and ability to adapt to different environments. Studying these miRNAs could reveal new insights into how these bees thrive [16,17,18]. In this study, we aimed to expand the foundational knowledge of gene regulation in stingless bees by focusing on the characterization of miRNAs in *M.quadrifasciata*. Understanding these regulatory pathways is essential not only for advancing basic science, but also for identifying novel strategies to promote the sustainability of bee populations, which are vital for food security and the stability of natural ecosystems.

## 2. Results

### 2.1. Overview of Melipona quadrifasciata Small RNAs

**Figure 1:**
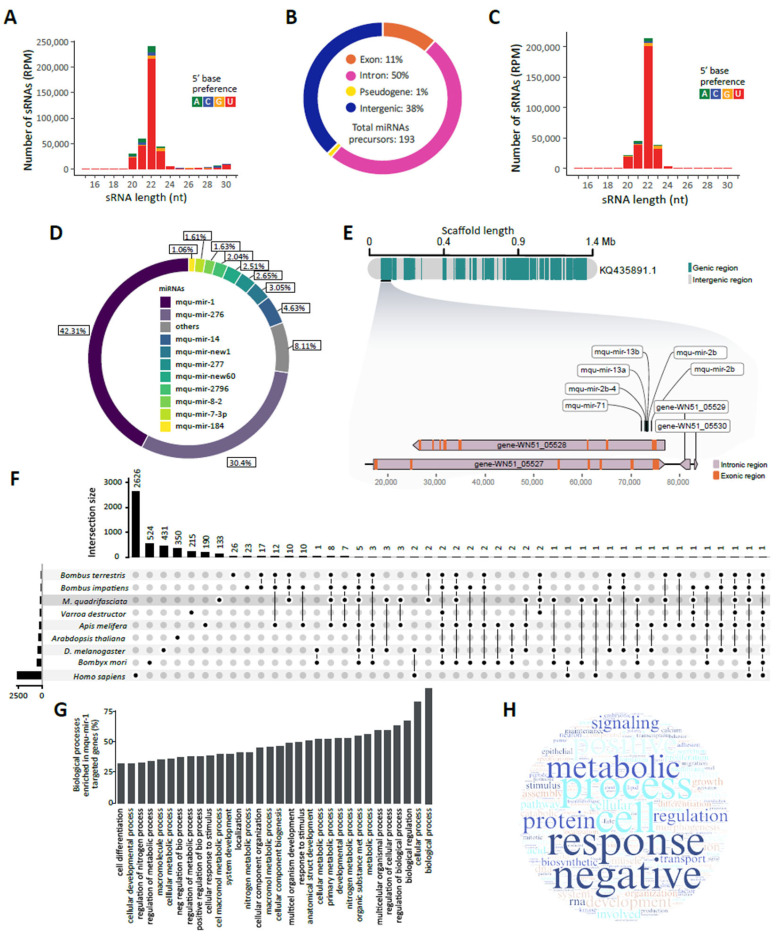
Overview of small RNAs and miRNA characterization in *Melipona quadrifasciata*. (**A**) Size distribution of small RNAs (15–35 nt) mapping to *M. quadrifasciata* genome. Colors represent the frequency of each 5′ nucleotide in mapped small RNAs. (**B**) Genomic origin of miRNA precursors identified in *M. quadrifasciata* according to features annotated on the reference genome. (**C**) Small RNA size profile of miRNA-derived small RNAs, indicating the size distribution of small RNAs originating from mature miRNAs; 5′ base preferences are indicated by color. (**D**) Relative contribution of the top 10 most abundant miRNAs to the total miRNA abundance. (**E**) Representation of largest miRNA cluster in *M. quadrifasciata*. In the scaffold, green represents genic regions while gray indicates intergenic regions. In the magnified region, miRNAs within the miRNA cluster are indicated by black flat lines. The genic context is also shown. (**F**) Overview of miRNA sharing among *M. quadrifasciata*, model organisms, and related species. Species sharing conserved miRNAs are connected by dots in the vertical line. Species-specific miRNAs appear as unconnected points. The upper bar graph shows the number of miRNAs that are either specific to or conserved across species. The left bar plot indicates the total number of miRNAs in the species analyzed. (**G**) General and (**H**) high-level GO biological processes related to genes predicted as targets of mqu-miR-1 in *M. quadrifasciata*.

A genome-based approach has previously demonstrated the existence of miRNA pathways in *Melipona quadrifasciata* [19]. However, we opted for a distinct strategy by employing the well-established miRDeep2 protocol. As a first step, to investigate the presence and profile of all small RNA (sRNA) populations in *M. quadrifasciata*, we aligned sRNA sequenced reads to the bee reference genome [20]. A peak at 22 nucleotides was prominently detected in the size distribution analysis, along with a selective accumulation of uracil (U) at the 5′ extremity (Figure 1A).

### 2.2. Small RNA-Based Identification and Characterization of miRNAs

Given the observed production of small RNAs, we subsequently examined the presence of microRNAs (miRNAs) within the small RNA populations of *M. quadrifasciata*. Through a small RNA-based analytical approach, we identified 193 high-confidence mature miRNAs. Examination of their precursor sequences revealed that 50% were derived from intronic regions, 38% from intergenic regions, 11% from exonic regions, and 1% from genomic regions containing pseudogenes (Figure 1B). The miRNA-derived small RNAs exhibited a size distribution ranging from 20 and 24 nucleotides long, with a prominent peak at 22 nt, and a strong enrichment of U at the first 5′ base (Figure 1C). We also conducted an analysis of individual microRNA (miRNA) abundance, identifying mqu-miR-1 and mqu-miR-276 as the most predominant elements (Figure 1D).

Next, we assessed miRNA abundance per scaffold, identifying KQ435794.1, KQ435727.1, and KQ435891.1 as the scaffolds with the highest miRNA content, containing 12, 9, and 6 miRNAs, respectively (Supplementary Figure S1A). Interestingly, the largest miRNA cluster (six miRNAs) was found in the scaffold KQ435891.1 (Figure 1E). Of the total miRNAs identified, 71 showed significant similarity to previously deposited miRNAs in miRBase, while 122 were likely *M. quadrifasciata*-specific elements (Supplementary File S1).

### 2.3. Analysis of miRNA Conservation

*M. quadrifasciata* miRNAs were previously identified using a genome-based prediction strategy [19]. Comparison between datasets revealed 87 shared sequences, while our analysis identified 106 novel candidates not previously described. Conversely, 113 sequences were exclusive to the earlier prediction (Supplementary Figure S1B). Evaluation of the evolutionary conservation of *M. quadrifasciata* miRNAs showed that *M. quadrifasciata* miRNA had greater similarity with those from other bees, particularly *Bombus terrestris* (33 shared miRNAs), *Bombus impatiens* (38 shared miRNAs), and *A. mellifera* (17 shared miRNAs), reflecting phylogenetic proximity within the Hymenoptera clade. Conservation was also observed with more distantly related species, including *D. melanogaster* (12 shared miRNAs), *Bombyx mori* (11 shared miRNAs), *Varroa destructor* (4 shared miRNAs), and *Homo sapiens* (2 shared miRNAs) (Figure 1F).

### 2.4. miR-1 Target Prediction and Gene Ontology

Next, to investigate a possible conserved role of mqu-miR-1, we evaluated the function of its putative target genes (Supplementary File S3, sheet 1). Functional annotation of the predicted target genes revealed enrichment in several Gene Ontology (GO) categories, including cell, protein binding, metabolic process, response to stimulus, negative regulation, and positive regulation. Additional terms such as morphogenesis, differentiation, and transport were also represented, suggesting that mqu-miR-1 may be involved in regulating a wide array of biological processes (Figure 1G and Supplementary File S3, sheet 2).

## 3. Discussion

The stingless bee *Melipona quadrifasciata* plays a crucial role in native pollination networks, with ecological and agricultural relevance [21]. Despite their importance, no miRNAs specific to Meliponini bees are currently cataloged in public databases. Most genetic studies of these species have focused on microsatellites for assessing diversity and conservation [22,23]. While transcriptomic analyses exist—mainly for *Melipona scutellaris*—they typically address gene expression under stress rather than miRNA identification [24,25]. Developing a comprehensive miRNA catalog for stingless bees is essential, as miRNAs serve as sensitive biomarkers of environmental stress and provide insights into gene regulation, which will aid future conservation and management strategies [26].

Analysis of the small RNA population in *M. quadrifasciata* provided valuable insights into its gene regulatory landscape. By mapping sRNA sequencing reads to the reference genome, we observed a size distribution characteristic of miRNA populations, including a strong enrichment of sequences around 22 nucleotides and a preference for uracil at the first 5′ position—features commonly associated with miRNAs in bees and other insects suggesting the presence of a miRNA pathway in *M. quadrifasciata* [27,28]. Given the prominent 22 nt peak observed in the sRNA size distribution, we proceeded to characterize this population by focusing on microRNAs. The majority of these miRNAs were 22 nucleotides in length. We also examined their genomic origin and found that most were derived from intergenic and intronic regions, while a smaller proportion (11%) originated from exons. The genomic distribution of these miRNAs may reflect differences in evolutionary constraints, with intergenic miRNAs potentially representing fast-evolving elements shaped by natural selection [29]. Interestingly, the largest miRNA cluster (six miRNAs) was found in KQ435891.1 (Figure 1E), a phenomenon previously observed in other insects [19].

The identification of miRNA clusters in the *M. quadrifasciata* genome, such as the six miRNAs located on scaffold KQ435891.1, aligns with observations in other insect species and suggests potential functional coordination. In insects like *D. melanogaster* and *A. mellifera*, miRNA clusters are frequently observed and are often transcribed as polycistronic primary transcripts from a single promoter [30,31,32]. This co-transcription mechanism typically leads to the coordinated expression of clustered miRNAs, which in turn often target genes involved in the same biological pathway or developmental process [33]. For instance, specific clusters in *Drosophila* have been shown to play crucial roles in developmental timing and tissue specification by synergistically regulating multiple targets within a network [34]. The evolutionary conservation of some miRNA clusters across different insect lineages also points to their fundamental importance [35]. Therefore, the clusters identified in *M. quadrifasciata* may represent groups of co-regulated miRNAs that act in concert to fine-tune complex biological processes, such as caste differentiation, stress response, or social behavior. Future studies aimed at characterizing the expression patterns of these clustered miRNAs and identifying their collective target genes could provide significant insights into their coordinated roles and evolutionary significance in stingless bees.

Among the miRNAs identified in *M. quadrifasciata*, two stood out due to their high abundance: mqu-miR-1 and mqu-miR-276, which together accounted for over 70% of total mapped reads. This prominent expression suggests central regulatory roles for both miRNAs in this species. mqu-miR-1 alone represented 42.3% of reads and has been previously reported in other insects, including *M. sexta*, *B. mori*, and *Dinoponera quadriceps* [36,37,38]. In bees, mqu-miR-1 is associated with reproductive processes and shows high expression in activated ovaries [39], suggesting a conserved role in development and reproduction. Similarly, mqu-miR-276 (30.4% of reads) may also play a key regulatory role. In other insects, mqu-miR-276 is involved in growth, hormonal signaling, circadian rhythms, and metabolic regulation (e.g., insulin signaling in *Drosophila*, embryogenesis and JH response in *Locusta*, and reproductive trade-offs in mosquitoes) [21,22,23]. These findings point to a potentially broad functional repertoire for miR-276 in stingless bees, possibly linked to development, reproduction, or stress response.

Overall, a great number of miRNAs described in this study have been characterized as regulating factors of reproductive behaviour, cast differentiation, and fundamental cellular machinery of growth and development in other insects, which suggests a similar role in *M. quadrifasciata,* mostly because these are deeply rooted across insect species [40,41,42,43]. Interestingly, we also found miRNAs that are involved in immune and stress responses. Specifically, these included miR-137, miR-375, miR-927, and miR-981, which are also present in *D. melanogaster,* where they influence the Toll immune pathway, metabolism during pathogen infection, metabolic homeostasis, and starvation resistance [44,45,46]. On that note, we highlight that understanding how such miRNAs influence bee health and responses to environmental stress might help understand key molecular factors in bee resistance to pesticides, global warming, and parasites. This could lead us to develop tools to improve bee resilience, guide safer agricultural practices, and support the long-term survival of these essential pollinators.

Previously, 87 *M. quadrifasciata* miRNAs were predicted using a genome-based approach [19]. In comparison, our sequencing-based strategy revealed a broader repertoire, identifying 106 novel miRNAs within a total of 193 candidates. Among the total identified, 71 showed sequence similarity—either complete or within the seed region—to miRNAs deposited in miRBase, suggesting functional conservation with miRNAs from other insects. The remaining 122 appear to be specific to *M. quadrifasciata*, highlighting the value of deep sequencing for uncovering species-specific regulatory elements. Considering that the previous study relied solely on a genome-based prediction strategy, it is important to note the limitations of such an approach, including the possibility of methodological artifacts [19]. While informative, genome-only analyses fall short in capturing the full complexity of small RNA populations and the actual products generated by RNA interference (RNAi) pathways under specific biological conditions. These methods are prone to high false-positive rates, as millions of genomic loci have the potential to form hairpin-like structures, yet only a small fraction are truly transcribed and processed through the miRNA biogenesis machinery [47]. In contrast, our strategy—based on total small RNA sequencing and leveraging the miRBase framework for the identification of both known and novel miRNAs—offers a more accurate and biologically relevant view of miRNA expression in vivo [47]. Our results revealed an overlap of 87 miRNA sequences between our dataset and the previous genome-based predictions, but also a substantial number of unique candidates in each. This divergence likely reflects both methodological differences and biological variation, as some genome-predicted miRNAs may not be expressed under the physiological conditions of the bees sampled in our study, and thus remained undetected in our sequencing data.

The presence of miR-1 as one of the most abundant and conserved miRNAs in our dataset aligns with its well-established role in muscle development and function across metazoans [48]. The high sequence conservation observed among insect homologs further supports a fundamental regulatory role maintained through evolution. The enrichment of GO terms such as cell, protein binding, metabolic process, and response to stimulus among its predicted targets suggests that miR-1 may regulate core cellular functions beyond muscle-specific pathways. Notably, the identification of terms related to morphogenesis, differentiation, and transport indicates a broader involvement in developmental processes, potentially including tissue remodeling and signaling events. These findings are consistent with previous studies demonstrating that miR-1 participates in regulating genes involved in cellular growth, differentiation, and homeostasis. Although our data provide new insights into miR-1 function in *M. quadrifasciata*, further experimental validation will be essential to confirm its specific regulatory roles and biological impact in this species.

Despite the inclusion of a limited number of individuals (*n* = 6), the miRNA profile identified in this study offers a valuable foundational dataset for future research on stingless bees. We acknowledge that the modest sample size and the absence of biological replicates represent limitations. These constraints, primarily due to limited funding and sample availability, necessitate caution in generalizing these initial findings and highlight the need for subsequent studies with larger cohorts and replicated experimental designs to validate and expand upon this preliminary miRNA repertoire.

## 4. Materials and Methods

### 4.1. Sample, Library Preparation, RNA Deep Sequencing, and Pre-Processing

Six whole *Melipona quadrifasciata* adult workers, all confirmed as healthy and negative for pathogenic viruses (according to the virus characterization pipeline described by Ferreira et al., 2025 [49]) were manually collected from an apiary located in Viçosa, southeastern Brazil, and their total RNA was individually extracted. Extraction was performed using TRIzol (Invitrogen, Carlsbad, CA, USA), following the manufacturer’s protocol. Bees were placed in 1.5 mL screw-cap tubes with 1.4 mm ceramic beads and ice-cold TRIzol, and homogenized using a Precellys Evolution system (3 cycles, 6500 rpm, 20 s, Bertin Technologies, Montigny-le-Bretonneux, France). Glycogen (10 µg, Ambion, Austin, TX, USA) was added to aid RNA pellet visualization. Samples were pooled, resuspended in RNase-free water, and stored at –80 °C. Small RNA libraries were prepared using the NEBNext Multiplex Small RNA Library Prep Set (New England Biolabs, Ipswich, MA, USA), with a modified 5′ adapter containing six additional nucleotides at the 3′ end to enhance barcode precision. Sequencing was performed on an Illumina HiSeq 4000 platform at the GenomEast facility (IGBMC, Strasbourg, France). Raw reads produced by RNA deep sequencing were submitted to adaptor trimming using the program Cutadapt [50]. Sequences with a low Phred score (<20), containing ambiguous nucleotides, and/or with lengths shorter than 15 nt were removed. The remaining reads, referred to as pre-processed, were used for posterior analyses.

### 4.2. Characterization of miRNA Populations and Genomic Analysis

Pre-processed reads were aligned with the *M. quadrifasciata* reference genome (GCA_001276565.1) using Bowtie [51], allowing up to one mismatch. The size distribution of mapped small RNAs (15–35 nt) was generated using in-house Perl scripts (v5.22.2) visualized in R. A genome-based approach previously demonstrated the existence of miRNA pathways in *M. quadrifasciata*. However, we opted for a gold-standard strategy by employing the well-established miRDeep2 protocol [52], incorporating all insect miRNAs available in miRBase v22.0 (downloaded in May/2024). Predicted miRNAs were manually curated following criteria adapted from Fonseca et al. [53], including: (i) the presence of a hairpin-like secondary structure; (ii) a hairpin length between 60–80 nt; (iii) a 2–3 nt 3′ overhang between mature and star sequences; (iv) ≥70% of reads with the same 5′ end; (v) a size distribution peak between 20–24 nt; and (vi) no overlap with annotated non-coding RNAs or repetitive elements. Candidates not meeting at least three of these criteria were discarded. Predicted miRNAs are detailed in Supplementary File S1. The genomic context of predicted precursors was determined using BEDtools [54] (intersect function) against the genome annotation. For cluster analysis, only miRNAs within 5000 nt of each other were considered. Overlapping miRNAs were excluded from further analyses.

### 4.3. miRNA Conservation

Mature miRNA sequences predicted in this study that did not match previously described insect miRNAs were aligned against all precursor sequences available in miRBase v22 using BLASTn online platform (available at https://blast.ncbi.nlm.nih.gov/Blast.cgi) to identify potential homologies. To compare prediction methodologies, BLAST online platform was also used to align the 193 miRNAs identified by miRDeep2 with the 200 pre-miRNAs predicted using the genome-based approach described by Araujo et al. (2024) [19], whose methodology was based on genome alignment, in contrast to ours, which relied on small RNA sequencing. This alignment allowed us to identify which miRNAs were shared between the two approaches and which were unique to each method. Additionally, to evaluate the evolutionary conservation of *M. quadrifasciata* miRNAs, we compared our dataset with miRNAs reported in other bee species and selected model organisms, including *D. melanogaster*, *H. sapiens*, *A. thaliana*, *B. mori*, and *V. destructor*.

### 4.4. Target and Functional Prediction of M. quadrifasciata miRNAs

To predict genes potentially regulated by *M. quadrifasciata* miRNAs, we employed in-house Python v3.13 scripts to extract putative 3′ untranslated regions (3′UTRs) from the publicly available genome assembly (GCA_001276565.1). Given current annotation limitations, the script retrieved 1000 nucleotides downstream of each gene’s annotated end coordinate to approximate intergenic 3′UTR regions. These sequences, along with the identified miRNAs, were submitted to miRNAconsTarget from sRNAtoolbox, which integrates TargetSpy [55], miRanda, and PITA. Only targets consistently predicted by all three tools were retained for downstream analyses. The predicted proteome was functionally re-annotated with EggNOG (e-value < 1 × 10^−10^; Supplementary File S2). A word cloud depicting high-level Gene Ontology (GO) biological process terms was then created using wordclouds.com with default settings.

## 5. Conclusions

This study provides the first comprehensive characterization of miRNAs in *Melipona quadrifasciata* using small RNA deep sequencing and genome-guided prediction. We identified 193 miRNAs, including 106 novel sequences not previously reported, expanding the current repertoire of known miRNAs in stingless bees. Expression profiling revealed that mqu-miR-1 and mqu-miR-276 dominate the miRNA landscape, suggesting potential roles in reproduction, development, and metabolic regulation. Comparative and evolutionary analyses demonstrated phylogenetic consistency, with greater conservation of miRNAs among closely related bee species. Finally, target prediction and functional annotation suggest that these miRNAs may regulate genes involved in essential biological regulatory processes. Together, these findings contribute to our understanding of gene regulation in stingless bees and provide a valuable foundation for future studies on miRNA function and evolution in non-model pollinators. Next steps should focus on functionally validating dominant and novel miRNAs to understand their roles in key biological processes and unique ecological adaptations.

## Data Availability

The *M. quadrifasciata* small RNA dataset generated in this study is available in the NCBI Sequence Read Archive (SRA) under BioProject PRJNA1273092. The predicted miRNAs sequences are currently available in Supplementary File S1.

## Supplementary Materials

The following supporting information can be downloaded at: https://www.mdpi.com/article/10.3390/ncrna11030048/s1, Figure S1: Distribution of microRNAs across the *M. quadrifasciata* genome; Supplementary File S1: Predicted miRNA candidates identified from *M. quadrifasciata*; Supplementary File S2: Functional re-annotation of the *M. quadrifasciata* proteome; Supplementary File S3: Predicted miR-1 targets and enriched GO terms.
