# Supplementary material for: Insights into miRNAs of the Stingless Bee Melipona quadrifasciata
Source: Noncoding RNA. 2025 Jun 19;11(3):48. doi: 10.3390/ncrna11030048 (PMC12196327; doi:10.3390/ncrna11030048)
Supplement: Supplementary file 1 [file ncrna-11-00048-s001.zip › ncrna-3607560-supplementary.docx]

Supplementary Material

Insights into miRNAs of the Stingless Bee
*Melipona quadrifasciata*

Dalliane Oliveira Soares ^1,†^, Lucas Yago Melo Ferreira ^1,†^, Gabriel Victor Pina Rodrigues ^1^,
João Pedro Nunes Santos ^1^, Ícaro Santos Lopes ^2^, Lucas Barbosa de Amorim Conceição ^1^, Tatyana Chagas Moura ^2^, Isaque João da Silva de Faria ^2^, Roenick Proveti Olmo ^3^, Weyder Cristiano Santana ^4^, Marco Antônio Costa ^2^
and Eric Roberto Guimarães Rocha Aguiar ^5,^*

^1^ Department of Biological Sciences, Center of Biotechnology and Genetics,
Universidade Estadual de Santa Cruz (UESC), Ilhéus 45662-900, Brazil; dalli.biotec@gmail.com (D.O.S.); lucasmelobiomed@gmail.com (L.Y.M.F.); gvprodrigues.ppggbm@uesc.br (G.V.P.R.);
jpnsantos.bio@uesc.br (J.P.N.S.); lbaconceicao.ppggbm@uesc.br (L.B.d.A.C.)

^2^ Department of Biochemistry and Immunology, Universidade Federal de Minas Gerais,
Belo Horizonte 31270-901, Brazil; icarolopes.bio@gmail.com (Í.S.L.);
tatychagas1@gmail.com (T.C.M.); isaquejsf@gmail.com (I.J.d.S.d.F.); costama@uesc.br (M.A.C.)

^3^ Centre National de la Recherche Scientifique (CNRS) UPR9022, Inserm U1257, Institut de Biologie Moléculaire et Cellulaire, 16 CEDEX, 67084 Strasbourg, France; roenick@gmail.com

^4^ Department of Entomology, Universidade Federal de Viçosa, Viçosa 36570-900, Brazil;
weyder.santana@ufv.br

^5^ Postgraduate Program in Computational Modeling in Science and Technology,
Department of Engineering and Computing, State University of Santa Cruz (UESC), Ilhéus 45662-900, Brazil

***** Correspondence: ericgdp@gmail.com

^†^ These authors contributed equally to this work.

**
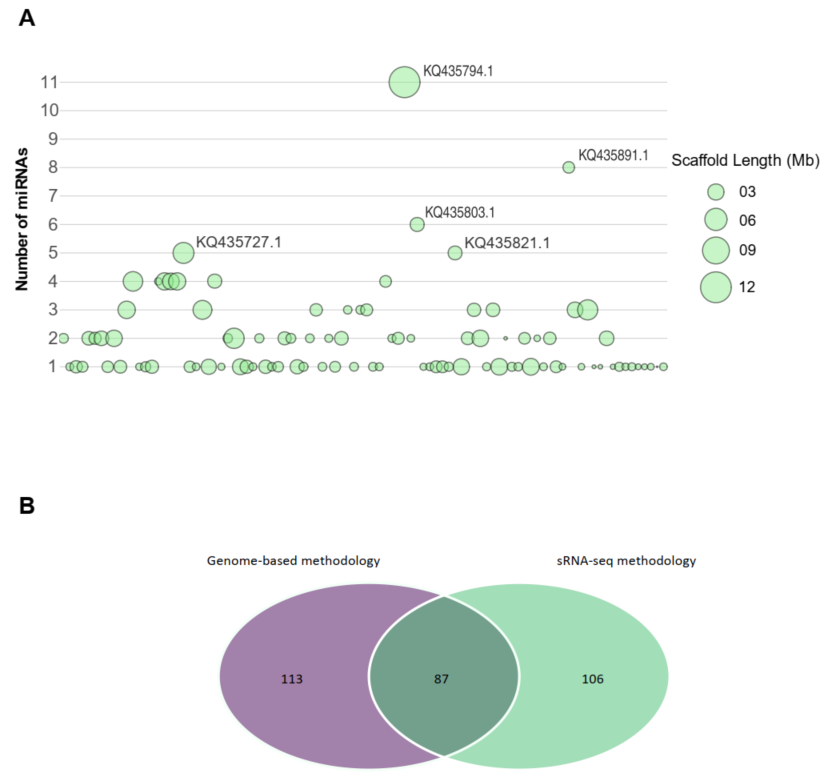
**

**Figure S1:** (**A**) Bubble plot displaying the number of miRNAs per scaffold of the *Melipona quadrifasciata* genome. Bubble size is proportional to scaffold length, and the Y-axis represents the number of miRNAs. (**B**) Venn diagram: in purple, the number of pre-miRNAs predicted by the genome-based methodology; in green, the number of miRNAs predicted by the sRNA-seq methodology; the intersection shows the miRNAs identified by both approaches.
